# The hepatocurative effects of *Cynara scolymus* L. leaf extract on carbon tetrachloride-induced oxidative stress and hepatic injury in rats

**DOI:** 10.1186/s40064-016-1894-1

**Published:** 2016-02-29

**Authors:** Emine Colak, Mehmet Cengiz Ustuner, Neslihan Tekin, Ertugrul Colak, Dilek Burukoglu, Irfan Degirmenci, Hasan Veysi Gunes

**Affiliations:** Department of Medical Biology, Faculty of Medicine, Eskisehir Osmangazi University, 26480 Eskisehir, Turkey; Department of Chemistry, Biochemistry Division, Faculty of Arts and Science, Aksaray University, Aksaray, Turkey; Department of Biostatistics, Faculty of Medicine, Eskisehir Osmangazi University, Eskisehir, Turkey; Department of Histology and Embryology, Faculty of Medicine, Eskisehir Osmangazi University, Eskisehir, Turkey

**Keywords:** *Cynara scolymus*, CCl_4_, Hepatocurative effect, DNA fragmentation, p53, Caspase 3

## Abstract

*Cynara scolymus* is a pharmacologically important medicinal plant containing phenolic acids and flavonoids. Experimental studies indicate antioxidant and hepatoprotective effects of *C. scolymus* but there have been no studies about therapeutic effects of liver diseases yet. In the present study, hepatocurative effects of *C. scolymus* leaf extract on carbon tetrachloride (CCl_4_)-induced oxidative stress and hepatic injury in rats were investigated by serum hepatic enzyme levels, oxidative stress indicator (malondialdehyde-MDA), endogenous antioxidants, DNA fragmentation, p53, caspase 3 and histopathology. Animals were divided into six groups: control, olive oil, CCl_4_, *C. scolymus* leaf extract, recovery and curative. CCl_4_ was administered at a dose of 0.2 mL/kg twice daily on CCl_4_, recovery and curative groups. *Cynara scolymus* extract was given orally for 2 weeks at a dose of 1.5 g/kg after CCl_4_ application on the curative group. Significant decrease of serum alanine-aminotransferase (ALT) and aspartate-aminotransferase (AST) levels were determined in the curative group. MDA levels were significantly lower in the curative group. Significant increase of superoxide dismutase (SOD) and catalase (CAT) activity in the curative group was determined. In the curative group, *C. scolymus* leaf extract application caused the DNA % fragmentation, p53 and caspase 3 levels of liver tissues towards the normal range. Our results indicated that *C. scolymus* leaf extract has hepatocurative effects of on CCl_4_-induced oxidative stress and hepatic injury by reducing lipid peroxidation, providing affected antioxidant systems towards the normal range. It also had positive effects on the pathway of the regulatory mechanism allowing repair of DNA damage on CCl_4_-induced hepatotoxicity.

## Background

The liver, the largest and most metabolically complex organ in the body, is involved in the metabolism of storage and biosynthesis (Hodgson and Levi 2004). It is also responsible for detoxification and metabolic homeostasis (Shin et al. 2013). Because the liver is metabolically active and the fundamental organ of biotransformation and the metabolism, it is influenced by reactive oxygen species (ROS) produced in liver cells as the by-products of normal metabolism and detoxification reactions. Accordingly, the liver has extensive antioxidant mechanisms to protect cells against ROS (Ellah 2011). However, the continuous and excessive production of ROS together with imbalances in endogenous antioxidant mechanisms causes oxidative stress. This stress induces damage to several biomolecules including lipids, proteins and DNA and, over time, contributes to liver injury. This injury is often differentiated as acute and chronic. Chronic liver disease has continuous formation from steatosis to chronic hepatitis, fibrosis, cirrhosis, and hepatocellular carcinoma (Coballase-Urrutia et al. 2011; Loguercio and Federico 2003; Vitaglione et al. 2005). As a result of the continuous development of cirrhosis, the hepatic functions of the liver are inhibited.

The plant *Cynara scolymus* L. (globe artichoke) is a pharmacologically important medicinal plant belonging to the Asteraceae family (Lattanzio et al. 2009; Mehmetçik et al. 2008); it is commonly grown in Mediterranean countries (Wang et al. 2003). Artichoke leaves contain phenolic acids, sesquiterpene lactones, flavonoids, phytosterols (taraxasterol), sugars, inulin, enzymes, and essential oils (Lattanzio et al. 2009; Wang et al. 2003; ESCOP 2003). The pharmacologically fundamental constituents of the leaf are phenolic acids and flavonoids. The phenolic acid derivatives include caffeoylquinic acids such as 3-caffeoylquinic acid, cynarin, and caffeic acids. The flavonoid compounds of the leaf are luteolin-7-β-D-glucoside, luteolin-4-β-D-glucoside and luteolin-7-β-rutinoside (Lattanzio et al. 2009; ESCOP 2003).

The leaf of the *C. scolymus* has been used for centuries as an antimicrobial, anti-inflammatory, choleretic, hepatoprotective, cholesterol-lowering, lipid-lowering, and glucose-lowering substance in Turkey, Southern Europe and Mediterranean countries (Lattanzio et al. 2009; Jimenez-Escrig et al. 2003). Several in vivo and in vitro studies have been conducted to examine the applications of *C. scolymus* leaves. As a result of these studies -in addition to the traditional usage- anti-carcinogenic, anti-apoptotic and anti-HIV characteristics were also determined (Miccadei et al. 2008; Robinson et al. 1996; Yang et al. 2001). The reason for the extensive usage of the leaf of the *C. scolymus* is described as being due to the additive and synergistic effects of the various compounds in the structure.

There have been several studies associated with liver pathology and the *C. scolymus* leaf extract. Gebhardt (1997) demonstrated the antioxidant and hepatoprotective effects of the *C. scolymus* leaf extract in primary cultured rat hepatocytes (Gebhardt 1997). Additionally, in a study with human leukocyte cultures, caffeic acid-, chlorogenic acid-, cynarin- and lutein-containing *C. scolymus* leaf extracts have been reported to have antioxidative effects, with the *C. scolymus* leaf extract expressing its antioxidant affects as a radical scavenger and a PMA-induced radical generation inhibitor (Pérez-García et al. 2000). Furthermore, as a result of in vivo studies in rats, the *C. scolymus* leaf extract has been suggested to reduce lipid peroxidation (Speroni et al. 2003) and protein oxidation as well as increase glutathione peroxidase activity (Jimenez-Escrig et al. 2003). Although there are several in vivo and in vitro studies regarding the hepatoprotective effects of the *C. scolymus* leaf extract, none of the studies describe its hepatocurative effects. The present study has been carried out to investigate the curative roles of the *C. scolymus* leaf extract against CCl_4_-induced oxidative stress and hepatic injury in rats. To determine and exhibit the oxidative stress after application of CCl_4_ and to evaluate the therapeutic effect of *C. scolymus* leaf extract; ALT and AST, the biochemical markers of liver damage, were measured. The severe hepatic damage caused by CCl_4_ toxicity was determined and also the therapeutic effects of the *C. scolymus* leaf extract were evaluated by measuring the level of the oxidative stress indicator MDA, the activities of endogenous antioxidants superoxide dismutase (SOD) and catalase (CAT). DNA fragmentation, p53 and caspase 3 levels were identified to determine the therapeutic effects of the *C. scolymus* extract on cell proliferation, apoptosis, DNA damage and homeostasis changes which were caused by CCl_4_ toxicity.

## Results

### *Cynara scolymus* leaf extract effects on serum hepatic enzyme levels

As shown in Table 1, the serum AST and ALT levels were significantly higher (P < 0.001) in the 0.2 mL/kg CCl_4_ group after the CCl_4_ administration, and significant decreases in the serum AST and ALT levels (P < 0.001) were obtained compared with the 0.2 mL/kg CCl_4_ group after the *C. scolymus* leaf extract (1.5 g/kg/day) application on the curative group. AST an ALT levels were decreased 37 and 49 % in the recovery group compared with 0.2 mL/kg CCl_4_ group. In the same duration, serum levels of AST and ALT were decrease by 40 and 52 % respectively in curative group compared with 0.2 mL/kg CCl_4_ group.

**Table 1:** Serum levels of AST and ALT

Groups (n = 7)	AST (U/l)	ALT (U/l)
Control	184.14 ± 5.78^+++^	70.71 ± 6.24^+++^
Olive oil	284.00 ± 32.10***^, +++^	117.71 ± 26.56***^, +++^
Cynara extract	248.42 ± 19.68***^, +++^	78.86 ± 16.23^+++^
0.2 ml/kg CCl_4_	342.14 ± 21.42***	201.00 ± 9.74***
Recovery	213.57 ± 10.45*^, +++^	101.00 ± 8.92***^, +++^
Curative	202.42 ± 21.57^+++^	96.43 ± 7.28***^, +++^

* P < 0.05, ** P < 0.01, *** P < 0.001 values are compared with the control group

^+^ P < 0.05, ^++^ P < 0.01, ^+++^ P < 0.001 values are compared with the CCl_4_ group

### *Cynara scolymus* leaf extract effects on the MDA levels, SOD and CAT activity of the liver tissue

The hepatocurative effects of *C. scolymus* leaf extracts on MDA levels, SOD and CAT activity of the liver tissue are shown in Table 2. MDA levels were increased significantly after the CCl_4_ application (P < 0.001). MDA levels were significantly lower after the application of the *C. scolymus* leaf extract in the curative group (P < 0.001). Moreover, there was a significant decrease in MDA found in the curative group versus the recovery group (P < 0.01). Administration of CCl_4_ decreased SOD activity (P < 0.001) significantly when compared to control group levels. Also significant decrease on the SOD activity of the recovery and curative groups were determined compared with both the control and 0.2 mL/kg CCl_4_ groups. The recovery group’s SOD activity decreased significantly versus the curative group (P < 0.001). SOD activity was decreased in the *Cynara* extract group without CCl_4_ application. This was an unexpected result but in this group MDA level was also significantly increased compared to control group and this demonstrated the lipid peroxidation that may be decreased the antioxidant activity of SOD. So that results need further investigation. Administration of CCl_4_ also significantly decreased CAT activity (P < 0.001) than that of the control group. After the application of the *C. scolymus* leaf extract, there was a significant increase (P < 0.001) observed in the curative group compared with both the recovery (P < 0.001) and 0.2 mL/kg CCl_4_ (P < 0.001) groups. This significant increase was also determined on the CAT level of the *C. scolymus* leaf extract group.

**Table 2:** MDA, SOD and CAT levels of liver tissues

Groups (n = 7)	MDA (nmol/g protein)	SOD (% inhibition)	CAT (kU/gr protein)
Control	0.36 ± 0.02^+++^	57.57 ± 1.13	2.89 ± 0.40^+++^
Olive oil	0.75 ± 0.04***^, +++^	55.43 ± 6.07***^, +++^	2.75 ± 0.19^+++^
Cynara extract	0.66 ± 0.02***^, +++^	33.57 ± 3.73***^, +++^	4.10 ± 0.70**^, +++^
0.2 ml/kg CCl_4_	1.04 ± 0.22***	46.57 ± 2.69***	1.43 ± 0.30***
Recovery	0.81 ± 0.16***^, +++^	31.00 ± 2.64***^, +++^	1.51 ± 0.49***
Curative	0.60 ± 0.08***^, +++, a^	38.57 ± 3.21***^, +++, b^	3.94 ± 0.37***^, +++, b^

* P < 0.05, ** P < 0.01, *** P < 0.001 values are compared with the control group

^+^ P < 0.05, ^++^ P < 0.01, ^+++^ P < 0.001 values are compared with the CCl_4_ group

^a^ P < 0.01, ^b ^P < 0.001 values compared between recovery and curative group

### *Cynara scolymus* leaf extract effects on DNA % fragmentation, p53 and caspase 3 levels of the liver tissue

The hepatocurative effects of *C. scolymus* leaf extracts on DNA % fragmentation, p53 and caspase 3 levels of the liver tissue are shown in Table 3. DNA % fragmentation, p53 and caspase 3 levels of the liver tissue increased significantly (P < 0.001) after the application of CCl_4_ compared with the control group. Treatment of CCl_4_ hepatotoxicity with the *C. scolymus* leaf extract decreased the DNA % fragmentation, p53 and caspase 3 levels significantly (P < 0.001). Compared with the recovery group, significant decreases of DNA % fragmentation (P < 0.01), p53 (P < 0.05) and caspase 3 (P < 0.001) levels of the liver tissues were obtained in the curative group. Our results also demonstrated that *C. scolymus* leaf extract application alone without CCl_4_ did not indicate any significant variation of DNA damage, p53 and caspase 3 levels.

**Table 3:** DNA fragmentation, p53 and caspase 3 levels of liver tissues

Groups (n = 7)	DNA fragmentation (%)	p53 (pg/ml)	Caspase 3 (ng/ml)
Control	20.11 ± 2.69^+++^	55.53 ± 0.93^+++^	5.03 ± 0.73^+++^
Olive oil	20.21 ± 0.82^+++^	53.34 ± 0.84^+++^	5.11 ± 0.85^+++^
Cynara extract	20.07 ± 1.83^+++^	55.90 ± 7.94^+++^	4.67 ± 0.29^+++^
0.2 ml/kg CCl_4_	33.96 ± 0.35***	112.39 ± 0.20***	7.42 ± 0.12***
Recovery	29.18 ± 2.61***^, +++^	71.74 ± 10.39***^, +++^	6.75 ± 0.29***
Curative	25.66 ± 2.15***^, +++, b^	61.96 ± 5.96^+++, a^	5.04 ± 0.13^+++, c^

* P < 0.05, ** P < 0.01, *** P < 0.001 values are compared with the control group

^+^ P < 0.05, ^++^ P < 0.01, ^+++^ P < 0.001 values are compared with the CCl_4_ group

^a^ P < 0.05, ^b ^P < 0.01, ^c ^P < 0.001 values compared between recovery and curative group

### *Cynara scolymus* leaf extract effects on histological changes of the liver tissues

**Fig. 1:**
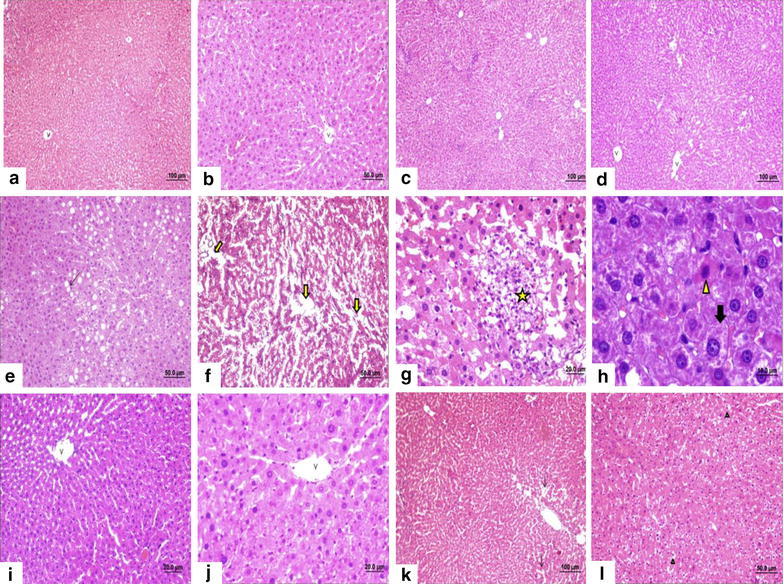
Histological investigations of the liver tissues. **a**, **b** Hemotoxylin and eosin-stained liver section of the control group (v vena sentralis, **a** X10, **b** X20). **c**, **d** *Cynara scolymus* leaf extract group (**c**, **d** X10). **e**–**h** CCl_4_ group; vacuolisation (**e** *thin arrow*, X20) sinusoidal dilation (**f** *arrow*), intense degeneration, nodular types of cellular damage (**g** *) necrotic cells and pyknotic nuclei of necrotic cells with eosinophilic cytoplasm (**h** *arrowhead*) a hypertrophic cell structures (**h** *black arrow*, X40,100). **i, j:** Recovery group (**i** X20, **j** X40). **k**, **l** Curative group; partial degeneration (*arrowhead*) and sinusoidal dilation (*arrow*) were examined (**k** X10, **l** X20)

The hepatocurative effects of *C. scolymus* leaf extracts on histological changes of the liver tissues are shown in Fig. 1. The liver tissue samples of the 0.2 mL/kg CCl_4_ group exhibited remarkable damage. Irregularities were observed in the parenchymal structure, and the classic lobular structure could not be distinguished. In addition, sinusoidal dilation (++), congestion (+), inflammation (++), intense degeneration (+++), vacuolisation, nodular types of cellular damage (+++), pycnotic nuclei of necrotic cells with eosinophilic cytoplasm (+++) and hypertrophic cell structures (+++) were observed. In the recovery group, sinusoidal dilation (+), inflammation (+), congestion (+) and cellular damage (+) were observed. Sinusoidal dilation (+) and congestion (+) were examined in the curative group.

## Discussion

The liver plays several important roles in the metabolism, including the biosynthesis of plasma proteins, gluconeogenesis and detoxification. Although the liver has strong regeneration capability, a specific amount of tissue loss prevents its ability to regenerate, and liver disease ensues (Xu et al. 2011).

Carbon tetrachloride (CCl_4_) is a well-known and widely used model for induced liver injury studies (Mir et al. 2010). CCl_4_ is a biologically inactive and stable substance that is chemically produced (Khan et al. 2012; Kose et al. 2005). It is transformed into reactive toxic metabolites by the cytochrome P-450 system in the liver. During this process, it is first converted to trichloromethyl by the cytochrome P-450 system. Then, in the presence of oxygen, it is rapidly transformed into trichloromethyl peroxide and chloroform, chemical configurations that have lost their hydrogen atoms. This free radical production overcomes the antioxidant defences of the liver and causes the oxidative destruction of cell membranes, resulting in serious liver tissue damage (Ajiboye 2010; Lima et al. 2007; Recknagel 1967) and one of the fundamental causes of CCl_4_ hepatotoxicity is the lipid peroxidative degradation of membranes (Srivastava et al. 1990). Damaged liver cells secrete liver specific enzymes into the bloodstream. These secreted enzymes, such as AST and ALT, are frequently used as biochemical indicators of liver damage and also play an important role in the investigation of the effects of therapeutic agents (Wallace and Meyer 2010; Mir et al. 2010; Lima et al. 2007). In the present study, the levels of AST and ALT were higher in the 0.2 mL/kg CCl_4_ group. The *C. scolymus* leaf extract (1.5 g/kg/day) application after the induction of liver injury by CCl_4_ resulted in decreased AST and ALT levels but there were no significant differences found between the recovery and curative groups. Although statistical sense was not obtained, in the same duration lower AST and ALT levels in curative group demonstrated that *C. scolymus* leaf extract accelerates the renewal mechanism of the liver. All these results indicated that there was a re-stabilization of the cell membranes and repair mechanisms in the hepatic tissue as a result of recovery against the oxidative stress more rapidly because of the in vivo antioxidant properties of the *C. scolymus* leaf extract. These results were also confirmed by MDA levels and histopathological evaluations in the current study. MDA, the end product of lipid peroxidation, is used as an oxidative stress indicator. In the present study, liver tissue MDA levels were higher in the 0.2 mL/kg CCl_4_ group after the induction of hepatocellular injury, and the *C. scolymus* leaf extract was found to decrease the levels of liver tissue MDA more than the recovery itself, presumably due to cell membrane repair.

In order to respond to oxidative stress, biological systems protect themselves with endogenous antioxidants such as catalase (CAT), glutathione (GSH), paraoxonase-1 (PON1), and arylesterase in the liver (Özbayer et al. 2011; El-Sayed et al. 2015). These antioxidants have important roles in the detoxification of free radicals and ROS resulting exposure of the various toxic chemicals and xenobiotics (Ajiboye 2010). The antioxidant GSH directly scavenges ROS and free radicals and as a part of the GSH redox system protecting biological systems from oxidative stress (El-Sayed et al. 2015). SOD, the endogenous antioxidant enzyme, catalyses the dismutation of the superoxide radical into molecular oxygen and hydrogen peroxide. CAT, an important antioxidant enzyme for the protection of the cells from oxidative damage caused by ROS, catalyses the reaction of hydrogen peroxide to water and molecular oxygen (Hsiao et al. 2001). Antioxidant enzymes are easily inactivated by lipid peroxides or reactive oxygen species (Coballase-Urrutia et al. 2011) and the decreases of these antioxidant enzymes in the cells cause failure in the defence and result in liver damage (Ajiboye 2010; El-Sayed et al. 2015). In addition, decreases of the GSH, SOD and CAT activity of the liver tissues after the application of CCl_4_ have been described previously (El-Sayed et al. 2015; Ajiboye 2010; Coballase-Urrutia et al. 2011; Cuciureanu et al. 2009). In the present study, we demonstrated that CCl_4_ induced a significant decrease the activity of SOD and it was also determined that this reduction continued in the recovery group that did not receive treatment after CCl_4_ administration. Treatment with the *C. scolymus* leaf extract elevated the decreased levels of SOD activity and markedly reduced the hepatotoxicity of CCl_4_ compared with both 0.2 mL/kg CCl_4_ and recovery group. In the present study, the significant depletion of CAT level in the 0.2 mL/kg CCl_4_ group and the significant increase of the activity of CAT in the curative group after the application of the *C. scolymus* leaf extract were also demonstrated. The application of the *C. scolymus* leaf extract increased the activity of CAT in the liver tissue towards the normal range in the curative group. These results suggested that as a result of CCl_4_ hepatotoxicity, endogenous antioxidant SOD and CAT activities would be decreased in cases where the external reinforcement was not supplied although liver has self-recovery mechanism and *C. scolymus* leaf extract attenuated CCl_4_-induced hepatotoxicity.

CCl_4_ application produced ROS and free radicals, capable of producing oxidative DNA damage. Reactive intermediates resulting from the transformation of CCl_4_ bind to DNA covalently, resulting in the oxidation of DNA (Lavanya et al. 2009). This oxidative damage causes the formation of DNA adducts, genetic mutations, strand breakage and chromosomal alterations (Mir et al. 2010; Khan et al. 2012). The formation of DNA damages affect homeostasis of the cells and induced signal transductions associated with apoptosis and cell proliferation. In response to DNA damage triggers the tumour suppressor gene p53 expression and the protein product of p53 blocks the cell cycle in the G_1_ phase for repairing the DNA (van Gijssel et al. 1997). When the damage is severe, it triggers the apoptosis. There is not only DNA damage but also cytochrome c is released from the mitochondria as a result of the oxidative damage and this pathway also activates the p53 and caspases (Campo et al. 2008). Our results showed that CCl_4_ induced high levels of DNA fragmentation; p53 and caspase 3 in the rat liver and the application of the *C. scolymus* leaf extract as a treatment decreased them more in the curative group than in the recovery group.

Several studies have reported that application of CCl_4_ lead to a variety of structural changes in the liver tissue such as sinusoid dilation, congestion, inflammation, intense degeneration, vacuolisation, nodular types of cellular damage, pyknotic nuclei of necrotic cells with eosinophilic cytoplasm and hypertrophic cell structures (Mehmetçik et al. 2008; Hsiao et al. 2001; Cuciureanu et al. 2009). In this study, we observed similar histomorphological changes in CCl_4_ group. Irregularities were observed in the parenchymal structure, and the classic lobular structure could not be distinguished. Severe sinusoidal dilation, congestion, inflammation, intense degeneration, vacuolisation, nodular types of cellular damage, pycnotic nuclei of necrotic cells with eosinophilic cytoplasm and hypertrophic cell structures were observed in this group. Following the CCl_4_ induced hepatic injury, the application of *C. scolymus* leaf extract led to a reduction of these damages more than that of the recovery group and mild sinusoidal dilation and congestion were detected in curative group.

All the achieved results demonstrated that *C. scolymus* leaf extract used against liver injury induced by CCl_4_ has antioxidant and anti-apoptotic properties. The effectiveness of the leaf extract against liver diseases is due to the phenolic acids and flavonoids. While the phenolic acids possess a good anti-oxidant activity against peroxyl and hydroxyl radicals, flavonoids act as hydrogen donors and metal ion chelators (Lattanzio et al. 2009).

## Conclusions

The present study indicated that the *C. scolymus* leaf extract reduced the lipid peroxidation, provided the affected antioxidant systems towards the normal range and had positive effects on the pathway of regulatory mechanism allowing repair of DNA damage on CCl_4_ induced hepatotoxicity. These results suggested that the *C. scolymus* leaf extract may be used as a pharmaceutical phytomedicine in the treatment of liver diseases.

## Methods

### Plant material

5 % cynarin containing, commercially available *C. scolymus* leaf extract powder extracted with 75 % ethanol was purchased from Kale Naturel Herbal Products Company, Ltd., Balikesir, Turkey.

### Chemicals and reagents

CCl_4_, 30 % hydrogen peroxide (H_2_O_2_), 1,1,3,3 tetraethoxy propane, 2-mercaptoethanol, ammonium molybdate, and n-butanol were purchased from Sigma Chemical Co. (St. Louis, MO, USA). Phosphoric acid (H_2_PO_4_), hydrochloric acid (HCl), potassium chloride (KCl), potassium phosphate (KH_2_PO_4_), xylene, potassium phosphate monobasic (Na_2_HPO_4_), sodium carbonate (Na_2_CO_3_), and thiobarbituric acid (TBA) were purchased from Merck KGaA (Germany). Polyethylene glycol tert-octylphenyl ether (Triton X-100) was purchased from Electron Microscopy Sciences (USA-Hatfield, PA). The SOD determination kit (19160; Fluka, Hannover, Germany) and Total Protein Liquicolor (10570; Human GmbH, Germany) were used. The remainder of the chemicals used were of a highly pure grade.

### Experimental animals

Forty-two Sprague–Dawley male rats weighing 267.83 ± 25.81 g were used for the study. All of the animals were kept under identical laboratory conditions, including temperature (22 ± 2 °C) and lighting (12: 12 h light: dark cycle). The rats were fed with standard laboratory chow and tap water ad libitum. All of the rats were allowed to acclimatize for one week prior to the experimentation. The study was carried out in accordance with the guidelines of the Eskisehir Osmangazi University Local Ethics Committee of Animal Experiments.

### Experimental design

After acclimatization, the animals were randomly divided into six experimental groups (n = 7 rats per group) including the control, olive oil, 0.2 mL/kg CCl_4_, *C. scolymus* leaf extract, recovery and curative groups. In order to establish a normal control, the control group rats received saline only (0.2 mL/kg i.p.) for 10 days and tap water orally for 2 weeks. For the vehicle control, the olive oil group rats received olive oil only (0.2 mL/kg) for 2 weeks. During the first 10 days of the study, the 0.2 mL/kg CCl_4_, the recovery and curative group rats were intraperitoneally injected with CCl_4_ at a dose of 0.2 mL/kg in 50 % olive oil solution twice daily to induce liver injury as previously described (Hülyam et al. 2005; Rikans et al. 1994). After the CCl_4_ administration, the 0.2 mL/kg CCl_4_ group rats were sacrificed to determine the extent of the liver injury. The curative group rats were given *C. scolymus* leaf extract orally for 2 weeks at a dose of 1.5 g/kg dissolved in tap water after the CCl_4_ administration. After the CCl_4_ administration, to investigate the long term effects of CCl_4_ and to evaluate the self-recovery mechanism of liver, rats in the recovery group did not receive any drugs or treatment for 2 weeks. The *C. scolymus* leaf extract (1.5 g/kg) was dissolved in tap water and applied orally to the *C. scolymus* leaf extract group rats for 2 weeks without any CCl_4_ administration. In the current study the basis of *C. scolymus* leaf extract dose in 1.5 g/kg determination depending on the specified studies about hepatoprotective effects reported the effect of *C. scolymus* extract at a dose of 1.5 g/kg was more prominent than that of lower doses (Mehmetçik et al. 2008; Küskü-Kiraz et al. 2010; Afifi et al. 2014). The rats were anesthetised with ether 24 h after the final treatments.

### Blood and tissue collection and preparation for assays

Blood was withdrawn from the rats under ether anaesthesia 24 h after the final treatments. Blood samples were collected from the inferior vena cava to the proper tubes. The blood was centrifuged at 2063 ×*g* for 15 min at 4 °C, and the serum was obtained to determine liver function.

After sacrificing the rats by bleeding, their livers were carefully dissected and cleared of extraneous tissue, and the samples were divided into two parts for separate uses. One part of the liver tissue was immediately transferred into 10 % formalin for histopathological investigation. The other parts were immediately stored at −80 °C to obtain homogenates for the analysis of SOD, CAT, MDA, DNA fragmentation and protein determination. These samples were homogenised in potassium chloride (1 %) using an ultrasonic homogeniser. The liver tissue homogenate was centrifuged at 3 667 ×*g* for 15 min at 4 °C. The obtained supernatant was stored at −80 °C until the studies commenced.

### Measurements of ALT and AST in the rat serum

The serum levels of ALT and AST were determined by a Roche Modular P chemistry analyser (Roche Diagnostics, 9115 Hague Road, Indianapolis, IN 46250). Product test kits were supplied by Roche (Roche Diagnostics, 9115 Hague Road, Indianapolis, IN 46250).

### Protein determination

The amount of total protein in the liver tissue was determined spectrophotometrically by a total protein kit (Total Protein Liquicolor 10 570, Wiesbaden, Germany) designed in accordance with the Biuret Protein Assay method, and the resulting values were used for the levels of MDA and CAT activity in the liver tissue homogenates.

### Estimation of lipid peroxidation

Lipid peroxidation, in terms of MDA production (thiobarbituric acid-reactive species), was determined as previously described (Uchiyama and Mihara 1978). Briefly, 0.1 mL of liver homogenate samples, 3 mL of 1 % phosphoric acid, and 1 mL of 0.6 % 2-thiobarbituric acid were used. For a blank, 0.1 mL of distilled water was used. The mixture was boiled in a water bath for 45 min followed by cooling in ice and the addition of 4 mL of n-butanol to extract the cold thiobarbituric acid reactants. After cooling, 4 mL of n-butanol was added, and the samples were shaken. The butanol layer was separated by centrifugation at 2 808 ×*g* for 5 min. The optical density of the n-butanol layer was determined at 532 nm on a spectrophotometer (Shimadzu UV-1601 digital spectrophotometer, Schimadzu Corp., Kyoto, Japan). A standard curve of MDA was constructed using concentrations of 10, 8, 6, 4, 2, and 1 nmol. The lipid peroxidation activities are expressed as nmol/g of wet tissue for the liver tissue homogenates.

### Estimation of superoxide dismutase (SOD) and catalase (CAT) activity of the liver tissue

The SOD enzyme activity in the liver tissue samples was measured using a SOD determination kit (FLUKA, St. Louis, MO, Cat. No: 19160). The kit uses a highly water-soluble tetrazolium salt, WST-1 (2-(4-lodophenyl)-3-(4-nitrophenyl)-5-(2,4-disulfophenyl)-2H-tetrazolium, monosodium salt), which produces a water-soluble formazan dye upon being reduced by superoxide anions. Briefly, 20 μL of sample solution, 200 μL of WST-1 working solution and 20 μL of enzyme working solution were added to each well of a 96-well microplate and incubated at 37 °C for 20 min to produce a water-soluble formazan dye. The absorbance at 450 nm was measured using an enzyme-linked immunosorbent assay plate reader (Labsystems Multiskan EX; Thermo Labsystems, Finland, Vartaa, Finland). SOD activity (inhibition rate) was calculated using the formula {[(Ablank 1–Ablank 3)—Asample–Ablank 2)]/(Ablank 1–Ablank 3)} × 100.

The CAT activity of the samples was determined according to the method described previously (Goth 1991). Briefly, the supernatant was incubated in the substrate solution containing H_2_O_2_ (65 μmol per mL in 50 mmol/L phosphate buffer, pH 7.0) in a water bath at 37 °C for 60s. The enzymatic reaction was blocked by the addition of ammonium molybdate, and a yellow complex of hydrogen peroxide was obtained. The absorbance of this yellow complex was measured at 405 nm using the spectrophotometer.

### Determination of DNA fragmentation in the liver tissue

DNA fragmentation was determined using a spectrophotometric assay method based on the diphenylamine (DPA) reaction, which binds to deoxyribose (Hickey et al. 2001). In order to measure liver DNA fragmentation by spectrophotometry, 0.3 g of frozen liver was homogenised in a chilled lysis buffer (5 mmol/L Tris–HCl, 20 mmol/L EDTA, 0.5 % Triton X-100, pH 8). The homogenates were centrifuged at 26 000 ×*g* for 25 min to separate the intact DNA in the pellet from the fragmented DNA in the supernatant. The pellets were re-suspended in 0.5 mol/L perchloric acid. In order to reach a final concentration of 0.5 mol/L, 1917 µL of the supernatants were treated with 86 µL of concentrated perchloric acid. All of the samples were incubated at 90 °C for 15 min and centrifuged at 750 ×*g* for 10 min to obtain the supernatants. The supernatants and DNA standards at various concentrations were treated with diphenylamine and incubated for 18 h at room temperature. The absorbance of the samples and standards were measured at 600 nm. The percentage of DNA fragmentation was calculated using the formula fragmented DNA/(fragmented DNA + intact DNA).

### p53 levels in the liver tissue

The p53 pan ELISA kit was used for determine the levels of p53 protein (Roche Molecular Biochemicals, Germany, Cat. No: 11828789001). The p53 pan ELISA assay is based on the quantitative “sandwich enzyme-immunoassay” principle using two monoclonal antibodies directed against rat p53. Briefly, 1 g of weighed liver tissue samples was homogenized in ice cold RIPA buffer (low-salt) containing 20 mmol/L Tris, 0.5 mmol/L EDTA, 1 % Nonidet P40, 0.5 % sodium deoxycholate, 0.05 % sodium dodecyl sulphate, 1 mmol/L phenylmethylsulphonylfluoride, 1 µg/mL aprotinin, and 2 µg/mL leupeptin. The homogenate was centrifuged at 10 000 ×*g* for 10 min at 4 °C. The resulting supernatants were collected and diluted 1:5 with sample diluent to prepare samples. The samples were transferred to a streptavidin-coated microtiter plate with an anti-rat-p53 polyclonal antibody. p53 from the sample binds to the plate surface, and the peroxidase (POD)-conjugated detection antibody interacts with the immobilized p53. Following a washing step, the POD bound within the “sandwich” complex is detected with the colorimetric substrate tetramethylbenzidine and levels are spectrophotometrically determined at 450 nm with a microplate reader. Sample concentrations were determined from calibration curve and tissue p53 concentrations were expressed as pg/mL.

### Caspase 3 levels in the liver tissue

USCN Life Science Inc. caspase 3 ELISA kit (Cat. No: SEA626Ra) was used to measure caspase 3 concentrations in the liver tissues. Briefly, the liver tissues (1 g) were homogenized in ice cold PBS (0.02 mol/L, pH 7.0) and then centrifuged at 5 000 ×*g* at 4 °C for 5 min. Standards and samples were added to the appropriate microtiter plate wells with a biotin-conjugated antibody specific to caspase 3. Avidin conjugated to Horseradish Peroxidase (HRP) was added to each microplate well and incubated. After TMB substrate solution was added, biotin-conjugated antibody and enzyme-conjugated Avidin showed a change in colour. The enzyme-substrate reaction was terminated by the addition of sulphuric acid solution and the colour change was measured spectrophotometrically at a wavelength of 450 nm. The concentrations of caspase 3 in the samples were then determined by comparing the O.D. of the samples to the standard curve. Levels were expressed as ng/mL.

### Histopathological evaluation

The livers were routinely processed in 10 % formalin solution and embedded in paraffin. Tissue Sections (5 μm) were obtained and stained with haematoxylin and eosin (H&E). The histopathological examinations were performed under a light microscope (NIKON, Japan). All of the histopathological examinations were performed by a histologist who was blinded to all of the tissue specimens. Samples were evaluated as (+) less damaged, (++) moderately damaged, (+++) severely damaged.

### Statistical analysis

The normality of the continuous variables was tested with the Kolmogorov–Smirnov test. Normally distributed variables were compared between groups using a one-way analysis of variance (ANOVA) with Tukey’s test. All results are expressed as mean ± SD (standard deviation). P values less than 0.05 were considered significant. Data analyses were performed using SPSS version 15.0.

## Abbreviations

CCl_4_: carbon tetrachloride
MDA: malondialdehyde
ALT: alanine-aminotransferase
AST: aspartate-aminotransferase
SOD: superoxide dismutase
CAT: catalase
ROS: reactive oxygen species
